# ctDNA在非小细胞肺癌精准治疗中的临床前沿与应用进展

**DOI:** 10.3779/j.issn.1009-3419.2026.106.22

**Published:** 2026-07-20

**Authors:** Hongmin YU, Hua QIAO, Jie LI, Liyan DU

**Affiliations:** 066000 秦皇岛，秦皇岛市第一医院呼吸与危重症医学科; Department of Pulmonary and Critical Care Medicine, The First Hospital of Qinhuangdao, Qinhuangdao 066000, China

**Keywords:** 肺肿瘤, 循环肿瘤DNA, 液体活检, 分子残留病灶, 复发风险, 个体化治疗, Lung neoplasms, Circulating tumor DNA, Liquid biopsy, Molecular residual disease, Recurrence risk, Individualized treatment

## Abstract

循环肿瘤DNA（circulating tumor DNA, ctDNA）可通过微创、可重复的采血反映肿瘤负荷与分子演化，是非小细胞肺癌（non-small cell lung cancer, NSCLC）全病程精准管理的重要候选标志物。本文围绕检测策略、围手术期动态、根治性治疗后分子残留病灶（molecular residual disease, MRD）、复发预警及不可切除III期应用梳理最新证据。现有研究最一致地支持根治性治疗后ctDNA阳性与高复发风险有关，纵向持续阴性比单次阴性更能识别低风险人群；新辅助治疗期间ctDNA清除与病理缓解及长期结局有关，但不能替代病理或影像评估。表皮生长因子受体（epidermal growth factor receptor, EGFR）突变型NSCLC已有随机试验样本的MRD分析；间变性淋巴瘤激酶（anaplastic lymphoma kinase, ALK）或c-ROS肉瘤致癌因子-受体酪氨酸激酶（ROS proto-oncogene 1, receptor tyrosine kinase, ROS1）融合型早期患者已有小型前瞻性术后MRD数据，但证据基础仍有限；Kirsten大鼠肉瘤病毒癌基因同源物（Kirsten rat sarcoma viral oncogene homolog, KRAS）GTP酶突变型专门亚组研究仍不足。病理、影像与ctDNA不一致时，应优先复核检测质量并按复发部位风险完成定向影像或组织学确认，而不宜仅凭ctDNA改变标准治疗。ctDNA已具备较强临床有效性，但常规治疗决策仍需标准化分析性能、跨平台一致性及前瞻性临床效用验证。

## Abstract (en)

Circulating tumor DNA (ctDNA) enables minimally invasive, repeatable assessment of tumor burden and molecular evolution and is an important candidate biomarker for precision management of non-small cell lung cancer (NSCLC). This review summarizes current evidence on assay strategies, perioperative dynamics, molecular residual disease (MRD) after curative-intent treatment, relapse surveillance, and applications in unresectable stage III disease. The most consistent evidence shows that detectable ctDNA after definitive treatment is associated with a substantially increased risk of recurrence, whereas longitudinally undetectable MRD identifies lower-risk populations more reliably than a single negative result. ctDNA clearance during neoadjuvant therapy is associated with pathological response and long-term outcomes but cannot replace pathology or imaging. In oncogene-driven disease, epidermal growth factor receptor (EGFR)-mutated NSCLC has MRD evidence from samples collected in a randomized trial; a small prospective postoperative study has provided direct MRD evidence in early-stage anaplastic lymphoma kinase (ALK)- or ROS proto-oncogene 1, receptor tyrosine kinase (ROS1)-fusion-positive disease, although the evidence base remains limited; Kirsten rat sarcoma viral oncogene homolog (KRAS), GTPase specific postoperative MRD studies remain insufficient. When pathology, imaging, and ctDNA are discordant, assay validity should first be confirmed and lesion-directed imaging or tissue confirmation pursued according to the suspected relapse pattern; ctDNA alone should not trigger deviation from standard therapy. ctDNA has substantial clinical validity, but routine treatment decisions still require standardized analytical performance, cross-platform reproducibility, and prospective evidence of clinical utility.

肺癌仍是全球癌症死亡的主要原因，非小细胞肺癌（non-small cell lung cancer, NSCLC）约占肺癌的85%^[1]^。围手术期免疫和靶向治疗改变了可切除NSCLC的治疗模式，但同一病理分期患者的复发风险和治疗获益仍存在明显差异^[2]^。组织检测仍是病理诊断和分子分型的基础；组织不足或难以取材时，循环肿瘤DNA（circulating tumor DNA, ctDNA）可作为补充性分子检测手段^[3]^。早期和局部晚期NSCLC的核心问题已从“能否检出”转向“能否稳定识别分子残留病灶（molecular residual disease, MRD）并改善治疗决策”。现有国际建议认为，ctDNA-MRD具有较强临床有效性，但作为常规决策工具仍需前瞻性临床效用验证^[4]^。本文重点综述其风险分层、疗效评估、MRD监测及临床转化。

评价ctDNA研究应区分分析有效性、临床有效性和临床效用。前两者分别回答检测是否准确稳定、结果是否与复发或生存有关；临床效用则要求证明依据检测结果采取行动能够改善患者结局。当前NSCLC队列已较充分支持前两层，但真正改变治疗的随机或前瞻性干预证据仍有限。因此，本文同时关注阴性结果的可解释性、跨研究的异质性，以及从“风险标志物”走向“决策标志物”所需的证据。

## 1 ctDNA的生物学特征与检测策略

### 1.1 生物学脱落与可测分子数

ctDNA是循环游离DNA（cell-free DNA, cfDNA）中来源于肿瘤的部分，可携带体细胞突变、拷贝数改变、甲基化及片段特征。其血浆丰度受肿瘤体积、分期、转移部位和组织学类型等影响。早期NSCLC肿瘤负荷低，ctDNA常处于极低丰度；鳞癌通常较腺癌释放更多ctDNA，部分肺腺癌和单纯颅内复发则呈低脱落表型。因此，ctDNA阴性既可能代表低风险，也可能源于低脱落或检测灵敏度不足^[5,6]^。

cfDNA主要来自细胞凋亡、坏死及与细胞增殖有关的释放过程，清除速度较快，使ctDNA能够较为及时地反映肿瘤变化；但手术创伤、感染、炎症和剧烈运动也会增加非肿瘤cfDNA，从而稀释肿瘤来源信号^[7]^。对MRD而言，检测极限不仅由测序深度决定，更受血浆体积、cfDNA输入量、分子回收率和可追踪肿瘤位点数约束。若该次采血获得的血浆中未抽样到目标分子，单纯增加测序深度也无法克服这一抽样限制。因此，高质量报告应同时给出血浆量、有效cfDNA输入、独立分子数、检测下限及样本是否达到预设质控要求，而不应只报告“阴性”。

肿瘤脱落还具有空间差异。肝、骨或多器官进展通常比局限于肺内或中枢神经系统的复发更易在血浆中检出；早期肺腺癌、磨玻璃成分较多及部分驱动基因阳性肿瘤可长期保持低ctDNA水平。由此需要明确的重要临床边界是：阳性结果通常具有较高风险提示价值，而阴性结果的可信度必须结合基线是否可检出、组织学、复发部位倾向以及检测平台的有效分子覆盖共同判断。

### 1.2 技术路线、误差抑制与信号整合

临床研究主要采用肿瘤信息驱动型和肿瘤信息非依赖型策略。前者先测序肿瘤组织并追踪患者特异性变异，特异性较高，适合MRD；后者直接使用固定面板或全基因组信号，周转较快且不依赖组织，但需处理克隆性造血和低丰度假阴性。深度测序个体化肿瘤分析（cancer personalized profiling by deep sequencing, CAPP-Seq）建立了超敏ctDNA定量框架^[8]^；整合数字化误差抑制（integrated digital error suppression, iDES）通过分子条形码与背景校正降低噪声^[9]^。数字聚合酶链式反应（digital polymerase chain reaction, dPCR）适合已知少数热点的快速定量，但位点覆盖有限。跨平台评估显示，在低变异等位基因频率和低cfDNA输入条件下，不同平台一致性明显下降^[10]^。

近年来的核心思路由“把单个位点测得更深”转向“在有限分子中累积更多独立肿瘤信号”。MRDetect通过全基因组整合大量患者特异性变异提高超低丰度监测能力^[11]^；PhasED-Seq利用同一DNA片段上的相位变异降低随机错误^[12]^；机器学习引导的全基因组信号富集也可整合大量微弱肿瘤特征^[13]^。因此，检测下限不能脱离样本条件进行比较，研究更应报告特定cfDNA输入下的检出概率、空白限、检测限、定量限、重复性以及以患者为分析单位的敏感度和特异度。

极深测序也会放大克隆性造血背景。组织-血浆-白细胞配对分析显示，部分血浆变异来源于造血克隆而非肿瘤^[14]^，肿瘤蛋白p53（tumor protein p53, TP53）、DNA甲基转移酶3α（DNA methyltransferase 3 alpha, DNMT3A）、TET甲基胞嘧啶双加氧酶2（TET methylcytosine dioxygenase 2, TET2）等相关基因变异及老年患者尤其需要警惕。配对外周血白细胞测序、肿瘤组织一致性和变异生物学背景联合判读是降低假阳性的关键。美国临床肿瘤学会（American Society of Clinical Oncology, ASCO）与美国病理医师协会（College of American Pathologists, CAP）的联合综述^[15]^亦强调，ctDNA结果须在分析验证、临床情境和组织结果框架内解释。

### 1.3 多组学与分析前质量控制

突变之外，甲基化、拷贝数和片段组学可提供互补信号。多组学模型在早期肺癌及术后MRD中显示出提高检出性能的潜力^[16,17]^，片段长度筛选可富集较短的肿瘤来源cfDNA，并提高部分低丰度变异和拷贝数信号的可见度^[18]^。但模型性能高度依赖训练人群、批次校正和阈值锁定；若在同一数据集中反复选择特征并报告最优界值，容易高估泛化能力。因此，多组学检测策略进入临床应用前应完成独立外部验证，并明确各类信号对最终判定的贡献。

分析前流程可决定最终灵敏度。使用乙二胺四乙酸（ethylenediaminetetraacetic acid, EDTA）采血管时应按验证流程尽快分离血浆；细胞稳定管也须遵循运输温度和处理时限。双次离心、避免白细胞裂解、统一冻融次数及随访序列中的采血体积，有助于降低非生物学波动。cfDNA不足、建板失败或可追踪位点不足应作为检测失败单独报告，不能归入ctDNA阴性。

## 2 ctDNA在早期及可切除NSCLC中的应用

### 2.1 术前ctDNA：风险分层而非单独筛选

术前ctDNA检出率受分期、组织学和检测灵敏度共同影响，因而不同研究间差异较大。传统面板在I期肺腺癌中的灵敏度有限；TRACERx队列采用基于全基因组的超敏肿瘤信息驱动平台分析171例早期肺癌患者，在肺腺癌中术前检出率达到81%，I期肺腺癌中为53%，并显示极低水平ctDNA仍与较差结局有关^[19]^。这一结果说明，既往术前阴性人群中包含一部分低于常规检测下限的患者。

TRACERx研究^[20]^进一步显示，术前未检出ctDNA可识别生物学相对惰性的肺腺癌亚群，而鳞癌因ctDNA释放较高，阴性结果的含义并不完全相同。因此，术前ctDNA应与病理类型、肿瘤体积、淋巴结状态和驱动基因背景联合解释。目前尚无证据支持仅依据术前ctDNA阴性降低手术或围手术期标准治疗强度。

将ctDNA与定量影像结合可能比任一单独指标更接近真实风险。Tran等^[21]^在根治性治疗的早期NSCLC中发现，治疗前或治疗后ctDNA均与复发及总生存期（overall survival, OS）有关，联合影像肿瘤体积可进一步划分风险层级。另一项I-IIIA期可手术队列^[22]^也支持围手术期ctDNA的预后价值。这一整合策略尤其适用于“体积大但ctDNA阴性”或“体积小但ctDNA阳性”的不一致病例：前者需考虑低脱落和分析灵敏度，后者则应排除克隆性造血并警惕高侵袭性生物学。

### 2.2 新辅助治疗期间的ctDNA动态变化

与单一基线值相比，新辅助治疗前后的ctDNA动态变化更能反映治疗反应。NADIM II期研究^[23]^中新辅助化疗联合纳武利尤单抗后ctDNA不可检出与更长无进展生存期（progression-free survival, PFS）[风险比（hazard ratio, HR）=0.26]和OS（HR=0.04）有关；5年随访^[24]^继续支持治疗后低ctDNA水平的长期预后价值。LCMC3研究^[25]^亦观察到新辅助阿替利珠单抗后ctDNA下降与肿瘤缩小和主要病理缓解（major pathological response, MPR）有关。

CheckMate 816研究^[26]^中纳武利尤单抗联合化疗组术前ctDNA清除率为56%，高于单纯化疗组的35%，ctDNA清除与病理完全缓解（pathological complete response, pCR）及无事件生存期（event-free survival, EFS）有关。5年分析^[27]^显示，术前ctDNA清除者OS率为75.0%，未清除者为52.6%，但pCR的区分能力仍更强。因此，ctDNA清除可补充pCR/MPR，不能替代病理学终点。

小型前瞻性新辅助免疫研究^[28]^同样提示，治疗后ctDNA下降或清除与病理疗效及无复发生存有关。临床上应优先比较同一患者、同一平台和相近cfDNA输入条件下的纵向变化，避免用不同实验室或不同检测下限的绝对值定义“清除”。对于基线ctDNA本就不可检出的患者，不能定义为治疗后清除；此类患者的疗效仍应依赖影像、手术可切除性和病理评估。

新辅助场景常出现病理、影像与ctDNA不一致。影像缩小且ctDNA清除但病理仍有残留时，应以手术病理决定局部残留，不将分子阴性等同于pCR；pCR但ctDNA持续阳性则属于分子高风险信号，应进入后述复核与定向影像流程。现阶段这些不一致状态更适合作为风险分层和临床试验分层依据，不应据此跳过手术或自动升级术后治疗。

### 2.3 术后ctDNA-MRD：预后价值、组织学差异与检测轨迹

术后ctDNA阳性是目前证据最一致的应用场景。LUNGCA-1前瞻性多中心队列^[29]^纳入330例I-III期NSCLC，采用769基因面板检测围手术期ctDNA，结果显示术后MRD阳性与显著增高的复发风险有关（HR=11.1）。早期CAPP-Seq研究及后续队列^[30,31]^同样显示，根治性治疗后可检出的ctDNA具有较高复发特异性，并常早于影像学进展；Waldeck等^[32]^虽仅纳入33例患者，但术后1-2周仍为ctDNA阳性的4例患者随后均出现疾病进展。

患者特异性肿瘤信息驱动检测进一步强化了多位点追踪价值。Chen等^[33]^建立个体化术后监测方法，显示标志性和纵向ctDNA状态均可识别高复发风险。Wang等^[34]^在128例I-III期切除患者中整合425基因测序与组织克隆结构：术后7天阳性与复发有关（HR=3.90），纵向阳性风险更高（HR=7.59）；在复发患者中，ctDNA可在73.5%的患者中早于影像学识别复发，中位提前145天。多中心研究^[35,36]^和超深度个体化检测也重复观察到术后阳性和纵向阳性与复发有关。

这些研究共同表明，标志性时点和纵向监测回答的问题不同。标志性检测用于根治性治疗后一次风险分层，适合辅助治疗前的研究性决策窗口；纵向检测则识别持续阴性、持续阳性、清除和再转阳4种轨迹。持续阳性通常代表最高风险，阳性后清除提示风险下降但并非等同治愈，再转阳则需要排除短暂技术波动后警惕分子复发。研究若仅把“任一时点阳性”合并为一组，会掩盖不同轨迹的临床含义。

不同病理类型应分别解读。鳞癌通常脱落更多ctDNA；I期、磨玻璃成分较多或驱动基因阳性的肺腺癌可呈低脱落，单次阴性更易出现假阴性。前述TRACERx^[19]^提示肺腺癌术前阴性可识别低风险亚群，但不能外推至所有病理类型。未来研究应预设腺癌/鳞癌亚组，并报告分期、吸烟状态、驱动基因和复发部位。

纵向检测可部分弥补单次检测灵敏度不足。在261例I-III期NSCLC术后队列^[37]^中，纵向持续MRD阴性患者仅3.2%复发，阴性预测值为96.8%，这提示“持续阴性”比“某一时点阴性”更接近低风险状态，但阴性预测值取决于随访时长、采样频率和复发构成，不能据此停止影像学随访。

### 2.4 术后采样窗口与复核策略

术后过早采血会受到手术创伤引起的非肿瘤cfDNA升高影响，从而稀释ctDNA信号。前述围手术期研究采用的术后采样窗口并不一致，既包括术后数日的早期采样，也包括术后数周的复测窗口。因此，不宜把某个单一时间点视为适用于所有平台的绝对标准。更合理的做法是区分“早期生物学观察窗口”和“辅助治疗决策窗口”：前者可用于研究手术后快速清除，后者应在患者恢复、cfDNA背景下降且不延误标准治疗的前提下完成。

实践中可考虑在术后2-4周设置首个决策性MRD时点，并在辅助治疗前后及其后固定间隔重复检测；若研究采用术后数日的早期样本，应预设较高的样本失败或稀释风险，并用后续样本确认。任何新发低丰度阳性都应核对原始分子数、可追踪位点、白细胞对照和既往肿瘤谱；当结果将触发重大治疗改变时，宜用独立采血样本复核，而非仅重复分析同一管血浆。

采样频率应与临床目的匹配。过密采血会增加成本和偶然阳性，采血间隔过长则可能延迟分子复发信号的发现，从而缩短其相对于影像学复发的提前时间。试验应预先规定标志性窗口、允许偏差、影像同步范围及检测失败处理，并采用时间依赖模型或预设标志性时点（landmark）分析纵向结果，降低获得更多检测机会的长期生存者所造成的时间相关偏倚。

### 2.5 ctDNA动态变化与辅助化疗决策

术前阳性转为术后阴性通常提示风险下降，术后持续阳性或由阴转阳提示残留或复发风险。Qiu等^[38]^和Tian等^[39]^均观察到围手术期ctDNA动态与复发及辅助治疗结局有关，但多为观察性或探索性分析，治疗选择存在混杂。现阶段ctDNA可用于预后沟通和试验分层，不宜单独作为省略或强化辅助化疗的依据。

辅助化疗后仍可检出的ctDNA可识别持续高风险患者^[40]^。但“预测化疗获益”必须证明治疗与ctDNA状态存在交互，而非仅显示MRD阳性患者预后更差；只有在MRD分层下随机比较治疗策略，才能区分预后效应与预测效应。

### 2.6 辅助治疗后ctDNA：组织学与驱动基因亚组

完成辅助治疗后仍可检出ctDNA通常提示较高复发风险，治疗过程中由阳转阴则提示分子学反应。阴性结果需结合病理类型、复发部位倾向和检测下限解释，尤其不能把低脱落肺腺癌的单次阴性等同于无残留病灶。

驱动基因阳性NSCLC中，表皮生长因子受体（epidermal growth factor receptor, EGFR）证据最为成熟。Jung等^[41]^对278例I-IIIA期根治性切除EGFR突变NSCLC纵向监测显示，术后ctDNA阳性与较差结局有关，但总体检出率偏低。2025年ADAURA探索性事后分析^[42]^纳入220例患者，结果显示肿瘤信息驱动的MRD阳性较影像学无病生存期（disease-free survival, DFS）事件提前中位4.7个月，临床敏感度为65%、特异度为95%；36个月DFS/MRD无事件率在奥希替尼组和安慰剂组分别为86%和36%（HR=0.23）。多数奥希替尼组事件发生于停药后，提示MRD可用于研究停药后风险，但尚不能据此延长或缩短疗程。

Dong等^[43]^在71例I-III期EGFR突变NSCLC中开展观察性MRD监测，部分持续阴性患者停用辅助EGFR-酪氨酸激酶抑制剂（EGFR-tyrosine kinase inhibitors, EGFR-TKIs）；22例IB-III期停药患者2年DFS率为80.2%，但仍有复发且缺乏随机对照。该研究支持“监测-停药-再启用”路径的可行性探索，不能证明其不劣于标准疗程。

间变性淋巴瘤激酶（anaplastic lymphoma kinase, ALK）或c-ROS肉瘤致癌因子-受体酪氨酸激酶（ROS proto-oncogene 1, receptor tyrosine kinase, ROS1）融合型NSCLC的早期术后MRD证据长期较少，既往研究更多集中在晚期动态监测。晚期ALK阳性NSCLC中，Horn等^[44]^在76例患者中显示血浆可检出疾病相关变异，并可纵向追踪ALK抑制剂疗效和耐药；另一项纳入43例患者、343份纵向样本的研究^[45]^中，ALK相关变异动力学总体反映疾病进程，并在44%的患者中提供早期分子进展信号。ROS1阳性NSCLC中，血浆可检出多种ROS1融合及耐药变异，但疾病进展时融合检出灵敏度约为50%^[46]^。这些数据证明相关分子信号“可检测、可追踪”，但不能直接外推为早期术后治疗指导。

针对早期术后场景，Fu等^[47]^于2024年开展的前瞻性非干预研究纳入49例可手术、携带可靶向融合[ALK、ROS1、重排期间表达基因（rearranged during transfection, RET）和成纤维细胞生长因子受体1（fibroblast growth factor receptor 1, FGFR1）]、间质上皮细胞转化因子（mesenchymal-epithelial transition factor, MET）受体酪氨酸激酶第14号外显子跳跃或原发性MET扩增的NSCLC，分析43份肿瘤组织和111份围手术期血浆样本；标志性时点和纵向MRD阳性结果的阳性预测值分别为100%和90.9%，阴性预测值分别为82.4%和86.4%，可检出MRD与更高复发率及更短DFS显著关联。研究作者同时指出，基因融合和MET异常本身较少见，且在血浆样本中较难检出。该研究为融合/MET异常早期术后MRD提供了重要直接证据，但样本量较小且多种驱动事件合并分析尚不足以分别确立ALK或ROS1特异的治疗指导效用。KRAS GTP酶方面，前述多中心可切除NSCLC研究已将KRAS相关变异纳入术后ctDNA追踪，但专门针对KRAS亚型、共突变背景和复发部位开展的MRD研究仍较少。综合前述研究，ALK/ROS1/KRAS术后MRD证据相对不足，主要可能与融合事件低频、低丰度血浆中结构变异捕获困难、驱动阳性肺腺癌低脱落及中枢神经系统复发的血浆漏检、既往围手术期研究通常将不同分子亚型合并分析，导致特定亚组统计效能不足，以及不同靶点围手术期治疗证据成熟时间不同有关。相较之下，前述EGFR研究已形成随机试验样本的MRD分析和初步减量观察。未来试验应按驱动基因预设亚组、优先追踪多个克隆性位点，并统一脑影像和采样窗口。

### 2.7 病理、影像与ctDNA的整合判读

ctDNA最合理的临床定位是补充而非替代病理和影像。对于“pCR但ctDNA阳性”，首先应排除分析性假阳性：复核原始独立分子数、阳性位点、背景错误模型、配对白细胞结果及与肿瘤组织的克隆一致性，并在独立采血样本中确认。若多个肿瘤特异性位点持续阳性、由低到高连续上升或与原发肿瘤克隆高度一致，应将其视为分子高风险，而不能因pCR简单归为低风险；可根据既往分期和复发倾向行增强计算机断层扫描（computed tomography, CT）、正电子发射计算机断层显像/CT（positron emission tomography/CT, PET/CT）及必要时脑磁共振成像（magnetic resonance imaging, MRI）检查。影像仍阴性时宜短间隔重复ctDNA/影像或优先进入MRD触发的干预试验，但在随机证据形成前，不应仅凭ctDNA自动改变标准辅助治疗。

对于“影像可疑但ctDNA阴性”，处置优先级相反：血浆阴性不能排除低脱落、局部小体积或单纯中枢神经系统复发，也不能排除因采样时点过早、cfDNA输入不足或可追踪位点有限造成的假阴性。因此，应按影像学恶性概率完成病灶特异的增强检查、短间隔复查或可行时组织学取样，不能因ctDNA阴性延迟诊断或标准治疗。若影像和病理均无复发证据而ctDNA仅单个位点低丰度阳性，则优先复核样本质量并短间隔重采；若多位点一致阳性或连续升高，则提高定向影像优先级。不同评估手段的结果不一致时，应结合各自反映的生物学维度和技术限制综合解释，而不应简单判定某一指标“错误”。

## 3 ctDNA在不可切除III期NSCLC中的应用

### 3.1 同步放化疗期间的动力学

不可切除III期NSCLC的标准路径通常为同步放化疗（concurrent chemoradiotherapy, cCRT）后巩固治疗。此类患者基线ctDNA检出率较高，但放疗导致的细胞死亡和炎症可造成治疗早期信号波动，短暂升高不应脱离后续清除轨迹解释；Moding等^[48]^提示cCRT后ctDNA清除状态与巩固治疗结局有关。Pan等^[49]^对139例局部晚期患者进行多时点检测，显示cCRT后残留及清除动力学与PFS和复发模式有关；不可手术局限期队列亦观察到类似关联^[50]^。因此，可设置“基线-cCRT早期-cCRT结束-巩固治疗前”的连续框架，其中cCRT结束后的标志性状态通常较治疗早期单点更能反映残留风险。

### 3.2 放化疗后MRD与巩固治疗

前述Moding等^[48]^的探索性分析显示，cCRT后仍可检出ctDNA的患者预后较差；在该亚组中，接受巩固免疫治疗者的结局优于未接受者。Lebow等^[51]^在17例接受根治性放疗的I-III期NSCLC患者中观察到，个体化ctDNA-MRD可识别治疗后高风险人群。Jun等^[52]^对不可切除III期NSCLC的生物标志物分析显示，cCRT后ctDNA阳性与阴性患者24个月PFS率分别为29%和65%，巩固治疗期间持续阳性同样提示较差结局。上述研究主要证明风险分层，而非治疗选择。cCRT后阳性者是否需强化、联合新药或延长巩固治疗，以及持续阴性者能否缩短治疗，均需随机验证。纵向分析还应采用时间依赖Cox模型或预设landmark分析，以减少未亡时间偏倚：患者只有存活并持续随访至后续采样时点，才有机会被归入相应纵向轨迹组；若未妥善处理，可能人为夸大持续阴性的保护效应。

### 3.3 局部复发、远处转移与中枢神经系统局限

局部晚期NSCLC的复发模式复杂，包括照射野内复发、远处转移和中枢神经系统进展。ctDNA可能更敏感地反映全身微转移，而对局部小体积或单纯脑内进展的血浆灵敏度较低。因此，ctDNA阴性不能替代胸部影像和脑MRI；相反，持续或再转阳可用于提高定向影像的优先级，并结合原始突变谱判断是否为既往肿瘤克隆。

III期患者cCRT后残留可同时反映局部控制不足和远处微转移。未来试验宜将局部失败、脑转移和其他远处失败作为竞争风险分别报告，并探索ctDNA轨迹与复发部位的关联，而非仅使用综合PFS终点。

## 4 纵向ctDNA监测、复发预警与干预试验

### 4.1 从单点状态到4种轨迹

固定时间点检测可完成一次风险分层，纵向监测则可识别持续阴性、持续阳性、清除和再转阳4种轨迹。如前所述，TRACERx^[20]^中3-6个月一次的监测可在部分术后标志性检测阴性的患者中进一步识别复发，LUCID亦显示ctDNA可早于影像学提示复发；纵向持续阴性则具有较高阴性预测值。上述结果共同说明，连续轨迹比单次状态提供更多风险信息，但脑内或局部小体积复发仍可能漏检。

轨迹解释还应纳入信号幅度和位点一致性。若多个已知肿瘤克隆位点在连续样本中同步升高，这种“再转阳”，比单个位点一次越过阈值更可信；仅维持一个采样周期的短暂“清除”也不能等同于长期持续阴性。研究应报告首次阳性时间、阳性位点数以及与影像事件的间隔。

### 4.2 分子提前量不等于临床获益

纵向监测尚未解决的关键问题是“提前发现是否带来更好结局”。在影像学阴性而ctDNA转阳时立即启动系统治疗，可能延长治疗暴露并带来过度治疗；等待影像学确认又可能错过分子复发窗口。因此，分子提前量属于检测性能和自然史指标，只有提前干预改善OS、DFS、生活质量或减少治疗负担时，才能转化为临床效用。

CTONG 2201研究^[53]^采用前瞻性、多中心、单臂设计，计划在术后2次连续MRD不可检出的IB-IIIA期患者中省略辅助治疗并进行每3个月监测。该研究针对降阶路径提供了重要可行性数据，但单臂设计难以完全排除患者选择和低风险富集。更高层级证据仍需将MRD持续阴性患者随机分配至标准治疗与减量策略，并预设非劣界值、补救治疗和脑转移监测。

### 4.3 分子复发后的确认与行动

影像阴性、ctDNA新发阳性时可采用4步路径：（1）审核样本质量、白细胞对照、阳性位点和原始分子数；（2）在短间隔内重新采血确认；（3）根据肿瘤生物学和既往复发模式开展定向影像；（4）在影像仍阴性时优先进入ctDNA触发的干预试验。若患者出现症状或多个位点快速上升，则不应因等待固定复测窗口而延误常规诊疗。

干预试验可分为MRD阳性升级、持续阴性降阶和分子复发提前治疗3类，均应设置同期对照并盲法判读影像；除DFS/OS外，还应评价治疗暴露、严重不良反应、假阳性后检查、患者焦虑和成本，避免把“更早知道复发”误认为延长生存。

## 5 临床转化的关键问题

### 5.1 核心问题一：检测流程、分析性能与结果报告标准化

临床推广首先需要解决“同一份样本在不同实验室能否得到一致且可解释的结论”。标准化应覆盖采血管和处理时限、血浆量与cfDNA输入、白细胞对照、检测限与特异性验证、阳性位点数阈值、失败样本处理和报告格式；外部质量评估^[54]^显示，不同实验室的前分析和分析流程可显著影响ctDNA回收与变异检出，国际液体活检学会的最低技术要求为跨平台质量框架提供了基础^[55]^。用于MRD研究或临床讨论的报告至少应注明检测策略及是否依赖肿瘤组织、可追踪位点数和克隆性依据、相对治疗的采样时点、血浆体积和cfDNA输入、以患者为单位的阳性判定规则、当前样本可实现的检测限、白细胞过滤方式，以及阴性和失败结果的限制；仅给出“阳性/阴性”不足以判断结果可信度。

2026年亚洲胸部肿瘤研究组Delphi共识^[56]^强调前分析处理、性能阈值、采样时点、术语和报告指标的协调，并指出目前不稳定的敏感度不支持在临床试验外依据MRD阴性常规降阶辅助治疗。该共识与前述欧洲肿瘤内科学会（European Society for Medical Oncology, ESMO）建议方向一致：ctDNA-MRD当前可作为高价值研究工具和风险标志物，但在缺少临床效用证据时不应取代标准病理和影像路径。

### 5.2 核心问题二：证明ctDNA指导治疗能够改善结局

预后相关并不等同于治疗预测。前述ADAURA^[42]^和CTONG 2201^[53]^等研究分别提供了风险分层和降阶路径的探索性依据，但均不足以证明“依据ctDNA采取行动”能够改善患者结局。下一阶段试验应分别回答：MRD阳性强化或延长治疗能否改善DFS/OS，持续阴性患者减量或停药是否具有非劣效性，分子复发提前治疗是否优于影像复发后治疗。随机化应按分期、组织学、驱动基因和检测策略分层，并预设脑转移、ctDNA阴性但影像复发及检测失败的处理。升级、降阶和提前治疗试验还需明确ctDNA清除的终点属性、重复采血确认和影像盲审，并公开检测失败率与报告周转时间。

### 5.3 核心问题三：成本、周转时间与可及性

高灵敏度MRD检测涉及组织测序、个体化建板、重复采血和生物信息学分析，成本不能只按单次价格评估，还应计入影像、治疗暴露、假阳性后检查和集中检测物流。实施研究^[57]^提示，ctDNA进入常规肿瘤学需同时明确临床责任、周转时间、报销和质量监管；只有当检测结果能够对应明确、可执行且有证据支持的路径时，才可能产生净价值。

### 5.4 证据成熟度与当前应用边界

不同场景的证据成熟度并不相同。术前ctDNA主要用于补充分期和生物学风险，并为后续追踪建立基线；由于检出率受组织学和肿瘤体积影响，不能因单次阴性降低手术或标准围手术期治疗强度。新辅助治疗期间ctDNA清除可补充pCR/MPR、影像和可切除性评估，但不能替代病理终点或单独决定是否手术。术后标志性MRD阳性具有较一致的高风险提示价值，可用于风险沟通和临床试验富集，但临床效用证据尚不足以支持仅据此强化或省略辅助治疗；纵向持续阴性可提高低风险判断的可信度，再转阳则应在复核样本后触发定向影像，而不能凭一次低丰度阳性启动长期系统治疗。不可切除III期cCRT后阳性可用于识别巩固期高风险，尚不能决定巩固治疗的缩短、延长或更换。驱动基因亚组中，EGFR证据相对成熟，ALK/ROS1和KRAS仍应优先纳入分子分层的前瞻性研究。总体上，任何场景都不应以ctDNA替代病理诊断、标准分期或影像随访。

## 6 总结与展望

ctDNA已把NSCLC残留病灶的观察窗口从影像可见阶段前移至分子阶段。现有证据最稳健的结论是：根治性治疗后ctDNA阳性提示高复发风险，连续检测比单点更能描述风险轨迹；新辅助治疗期间ctDNA清除可补充pCR/MPR和影像；不可切除III期NSCLC中，cCRT后残留及巩固期持续阳性提示不良结局。EGFR突变型NSCLC已有随机试验样本的MRD分析；ALK/ROS1已有小型、富集融合阳性患者的术后MRD前瞻性研究数据，但分子亚组样本仍少；KRAS特异的术后MRD证据仍需加强。

未来突破的重点不应停留在追求更低的检测限，而应围绕3个可验证目标：（1）在规定血浆输入和分析前条件下实现以患者为单位、可复现的分析性能；（2）以统一术语和采样窗口证明跨人群临床有效性；（3）通过随机或严谨的前瞻性试验证明升级、降阶或提前干预能够改善生存、生活质量或治疗负担。现阶段，ctDNA应作为病理、影像和临床评估的互补层，优先用于具备标准化检测和明确行动方案的研究。只有把“可检出”转化为“可解释、可行动、可获益”，ctDNA-MRD才可能真正进入NSCLC常规精准治疗。
